# Integration of Mesoporous Copper Coordination Polymer in Electrochemical Platform for Detection of Fluoroquinolone Antibiotic in Biological and Food Samples

**DOI:** 10.1021/acsomega.5c07161

**Published:** 2025-10-21

**Authors:** Iare S. Ribeiro, Tatianny de A. Andrade, Tiago A. Silva, Jemmyson R. de Jesus

**Affiliations:** † Research Laboratory in Bionanomaterials, LPbio, Department of Chemistry, 28120 Federal University of Viçosa, Viçosa, Minas Gerais 36570-900, Brazil; ‡ Department of Chemistry, Federal University of Viçosa, Viçosa, Minas Gerais 36570-900, Brazil

## Abstract

Antibiotics are indispensable in both human and veterinary medicine. However, their extensive and indiscriminate use has raised serious environmental and public health concerns, particularly regarding the emergence of antibiotic-resistant bacteria. In this context, continuous monitoring of broad-spectrum antibiotics, such as fluoroquinolone class, is essential. This study reports the development of an electrochemical sensor based on a copper-based coordination polymer, [Cu­(C_8_H_4_O_4_)]_*n*_/CPE, for the detection of fluoroquinolone antibiotic (ciprofloxacin, CIP) in synthetic urine and egg samples. [Cu­(C_8_H_4_O_4_)]_*n*_ was synthesized via a solvothermal method, employing 1,4-benzenedicarboxylic acid as the organic ligand and copper salt as the metal source. The material was characterized by Fourier transform infrared spectroscopy (FT-IR), X-ray diffraction (XRD), scanning electron microscopy with energy dispersive spectroscopy (SEM-EDS), thermogravimetric analysis (TGA), and Brunauer–Emmett–Teller (BET) surface area analysis. Electrode modification was optimized by varying the modifier content, pH solution, and electrochemical technique. Optimization using differential pulse voltammetry (DPV) revealed ideal conditions at pH 6.0 with 20% (w/w) [Cu­(C_8_H_4_O_4_)]_*n*_. Under this condition, the sensor exhibited excellent linearity (*R* ^2^ = 0.992), recoveries ranging from 78 to 108%, and relative standard deviation (RSD) bellow 10% (*n* = 3). The limits of detection (LOD) and quantification (LOQ) were 0.5 and 1.7 μmol L^–1^ in synthetic urine and 3.0 and 10.1 μmol L^–1^ in egg sample, respectively. The [Cu­(C_8_H_4_O_4_)]_*n*_/CPE sensor demonstrated high sensitivity and accuracy, highlighting its potential as reliable tool for monitoring CIP residues in food products and biological samples, thereby contributing to food safety and public health.

## Graphical abstract

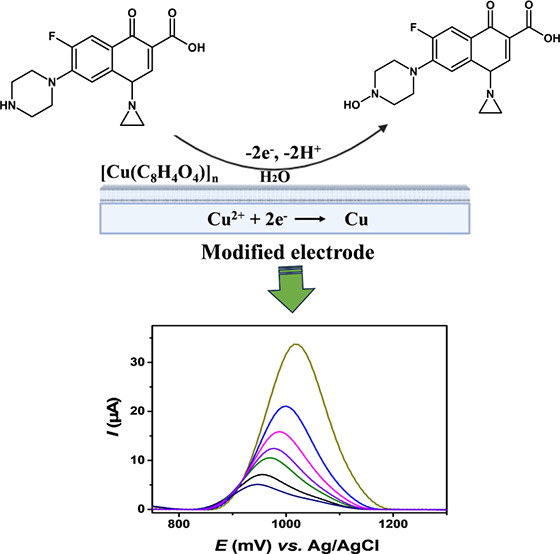

## 1 Introduction

Pharmaceutical drugs represent a broad spectrum of structurally complex organic molecules that are essential for the prevention and treatment of diseases in humans and animals.^1^ This group includes analgesics, antipyretics, antibiotics, antivirals, antiseptics, and anti-inflammatories.^2^ Among these substances, antibiotics represent a major segment of the global pharmaceutical market, not only due to their extensive therapeutic application but also due to their frequent occurrence in environmental samples.^3^ Within this class, fluoroquinolones (FQs) stand out for their broad-spectrum efficacy against pathogenic bacteria.^2^ Their use has expanded significantly in both human health and veterinary medicine, generating billions of euros in revenue. Between 2008 and 2019, the global FQ market generated approximately 501,938,402 euros in revenue.^4^ However, the increasing prevalence of antibiotic resistance has emerged as a critical global health concern, compromising the effectiveness of conventional antimicrobial therapies. Widespread and often indiscriminate use of antibiotics has played a central role in the emergence and dissemination of antibiotic-resistant bacteria.^5,6^ Resistance to quinolones, for example, has been reported since the clinical introduction of nalidixic acid. In the 1990s, ciprofloxacin (CIP) use in the United States increased by nearly 40%, coinciding with a marked rise in resistance among Gram-negative pathogens.^2^

In general, up to 70% of administered FQs are excreted unmetabolized, entering the environment mainly through domestic sewage.^2^ In addition, wastewater from pharmaceutical industry, hospital effluents, and agricultural runoffs represent significant sources of environmental contamination. The persistence of these compounds imposes selective pressure on microbial communities, thereby promoting the evolution and dissemination of multidrug-resistant bacteria.^7^

The use of FQs, such as CIPs, in veterinary and agricultural practice also raises serious concerns regarding food safety.^8,9^ Residual traces of CIP and its metabolites may persist in animal-derived products, including meat, milk, and egg.^8,9^ When these residues exceed established safety thresholds, they pose potential health risks to consumers. To address this issue, the European Union has set maximum residue limits (MRLs) of 100 μg kg^–1^ for CIP and its active metabolite in food products of animal origin.^10,11^

Several analytical techniques have been employed to monitor CIP in both biological and environmental samples.^12^ Chromatographic and spectrophotometric methods are widely used due to their accuracy and robustness.^12^ However, these approaches often require labor-intensive sample preparation, costly instrumentation, and generate considerable amounts of chemical waste.^12^ In contrast, electroanalytical techniques, such as voltammetry and amperometry, have gained attention as attractive alternatives due to their high sensitivity, rapid analysis, low operational cost, and suitability for miniaturization and on-site applications.^13^ In this context, the development of electrochemical sensors for CIP detection has attracted attention, employing diverse functional materials that are being explored to improve analytical performance.^14,15^

In this context, various modified electrodes have been developed for the electrochemical determination of CIP. For instance, Shafiei et al.^16^ reported a highly sensitive electrochemical sensor for CIP detection based on a carbon paste electrode (CPE) modified with silver decorated in polyoxometalate (POM), reduced graphene oxide (rGO), and an ionic liquid (IL). The integration of these components provided significant advantages, combining high conductivity, large surface area, low resistance, and unique properties of the nanocomposite. Cyclic voltammetry (CV) results revealed significantly enhanced electrocatalytic activity for CIP detection compared to other electrodes. The Ag@POM@rGO-IL/CPE exhibited a low limit of detection, a wide linear dynamic range, high stability, good reproducibility and repeatability, as well as a rapid response for CIP determination using the square wave voltammetry (SWV) technique.^16^

In another study, CPE modified with choline chloride (ChCl) was developed for the electrochemical determination of CIP. The modified electrode (ChCl/CPE) exhibited an electroactive surface area of 0.123 cm^2^, four times larger than that of the bare CPE. In addition to its simplicity, the sensor provided a lower detection limit and the widest linear range among recently reported CIP sensors. It also demonstrated excellent reproducibility and stability, with successful application in diverse matrices, including river water, eye drops, and egg samples, achieving satisfactory recovery rates.^17^ Meireles et al.^18^ also reported an electrochemical sensor based on cost-effective films, in which carbon black (CB) and silver nanoparticles (AgNPs) were embedded in a cross-linked chitosan (Ch) matrix, coated onto the surface of a glassy carbon electrode (GCE). This modified electrode (AgNPs-CB-Ch/GCE) showed good precision and was effectively applied for CIP detection in a synthetic urine sample, achieving recovery rates close to 100%.^18^

One class of material that has attracted significant attention in the development of electrochemical sensors, especially through electrode surface modification, is porous coordination polymers, commonly known as Metal–Organic Frameworks (MOFs).^19,20^ These hybrid materials, formed by the coordination of metal ions with multidentate organic ligands, exhibit exceptionally high surface area, tunable porosity, controlled chemical composition and abundant exposed active sites, making them highly suitable for integration into portable, sensitive and low-cost sensing devices. Additionally, the rational combination of metal centers with functionalized organic ligands can generate synergistic effects that enhance the electronic, redox and catalytic properties of the sensors.^21,22^ MOFs also demonstrate good stability under electrochemical operating conditions, ensuring long-term performance and reusability.^23–25^

In this context, the present study reports the synthesis of a copper-based mesoporous coordination polymer, [Cu­(C_8_H_4_O_4_)]_*n*_, for use in an electrochemical sensor for CIP detection in biological (urine) and food (egg) samples. Unlike previous sensors,^16,17^ that rely on additional conductive fillers or nanocomposites, our material, [Cu­(C_8_H_4_O_4_)]_*n*_, offers high surface area, structural tunability, and exposed active sites, which enhance sensitivity and selectivity. Its novelty lies in functioning effectively without the need for additional conductive materials or nanoparticles, thereby simplifying sensor fabrication while enabling efficient electron transfer and strong interaction with CIP, improving the analytical performance of the electrochemical platform.

## 2 Materials and Methods

### 2.1 Materials

Copper­(II) nitrate trihydrate 98% (Cu­(NO_3_)_2_ · 3H_2_O, CAS 10031-43-3) was purchased from Dinâmica (SP, Brazil). Sodium hydroxide 97% (NaOH, CAS 1310-73-2) was supplied by Isofar (RJ, Brazil). 1,4-Benzenedicarboxylic acid 98% (H_2_BDC, CAS 100-21-0), CIP 99%, uric acid 99%, glucose 97% (CAS 23403-54-5), enrofloxacin 98% (C_19_H_22_FN_3_O_3_, CAS 93106-60-6) were purchased from Sigma-Aldrich (MA, USA). Urea (CAS 57–13–6) was obtained from Dinâmica (SP, Brazil). Ascorbic acid 99% (CAS 50–81–7) was pushed from Vetec (RJ, Brazil). Calcium chloride 98% (CaCl_2_, CAS 10043–52–4) was purchased from Merck (HE, Germany), and sodium chloride 99% (NaCl, CAS 7647–14–5) from ACS Científica (SP, Brazil). All reagents were used without further purification. All reagents were used without further purification. All solutions were prepared using ultrapure water from a Milli-Q purification system (Millipore, Bedford, MA, USA). Unless otherwise stated, all other reagents were of analytical grade and used as received.

### 2.2 Synthesis of [Cu­(C_8_H_4_O_4_)]_*n*_

[Cu­(C_8_H_4_O_4_)]_*n*_ was synthesized using two-step procedure adapted from Jesus et al.^26^ Initially, deprotonation of H_2_BDC (3 mmol; 0.49 g) was achieved by dissolving the ligand in 15.0 mL of ultrapure water (18.00 MΩ cm), followed by the gradual addition of a NaOH solution (8.0 mol L^–1^). Subsequently, Cu­(NO_3_)_2_ (3 mmol, 0.72 g) was slowly introduced into the solution under continuous mechanical stirring for approximately 15 min to ensure complete homogenization (eq 1). The resulting mixture was transferred to a 25.0 mL PTFE (Teflon) vessel, which was sealed inside a stainless-steel autoclave and heated in an oven at 200 °C for 24 h under static conditions. After natural cooling to room temperature, the solid product was separated from the supernatant by centrifugation at 4000 rpm for 10 min. The precipitate was washed twice with ultrapure water to remove residual byproducts and then washed twice with ethanol to eliminate excess water and promote faster drying. Finally, the material was dried in an oven at 60 °C for 4 h and stored in a desiccator until use.

1 $$H_{2}BDC_{\left(aq\right)}+2NaOH_{\left(aq\right)}+Cu{\left(NO_{3}\right)}_{2\left(aq\right)}\to {[Cu\left(C_{8}H_{4}O_{4}\right)]}_{n\left(s\right)}+2NaNO_{3(aq)}+2H_{2}O_{\left(l\right)}$$

### 2.3 Characterization

Morphology and elemental distribution of the material were examined using Scanning Electron Microscopy coupled with Energy Dispersive X-ray Spectroscopy (SEM-EDS JEOL JSM-65LV, Tokyo, Japan), with an accelerating voltage ranging from 0.3 kV to 30 kV. Thermogravimetric Analysis (TGA) was performed to assess the thermal stability profile using a thermogravimetric analyzer (PerkinElmer, CT, USA) in a temperature range of 30 to 900 °C, at a heating rate of 10 °C min^–1^, under a nitrogen flow of 50 mL min^–1^. The diffraction pattern and structural purity were determined by powder X-ray diffraction (XRD) at room temperature, using a Rigaku diffractometer (Rigaku, Tokyo, Japan) with Bragg–Brentano geometry in continuous mode, within a 2θ range of 25 to 70° and CuKα radiation. Additionally, the Fourier Transform Infrared Spectroscopy (FT-IR) using a Bruker ALPHA II spectrometer (MA, USA) equipped with an ATR (Attenuated Total Reflectance) accessory, covering a range of 4000–400 cm^–1^. This procedure enabled the identification of the main absorption bands, providing information on the chemical bonds and functional groups present in the material. Raman spectroscopy was performed using a micro-Raman spectrometer (microRaman-In Via Renishaw, Gloucestershire, UK) configured in Stokes mode with a 785 nm excitation line. Raman mapping was employed, and each measurement was conducted with an integration time of 10 s per scan, accumulated over 50 scans to enhance the signal-to-noise ratio. The laser was focused on the sample at a Raman shift of 1100 cm^–1^, with the power set to 1% of its maximum to prevent material degradation. A 50× objective lens ensured precise focusing and high spatial resolution, enabling accurate data acquisition and reliable analysis. Finally, nitrogen adsorption–desorption isotherms of the material were obtained using a Nova 600 Series instrument from Anton Paar (Styria, Austria). The sample was degassed at 150 °C for 4 h to remove water and residual gases. The specific surface area was measured using the Brunauer–Emmett–Teller (BET) method, and the pore size distribution was determined using the Barrett–Joyner–Halenda (BJH) method.

### 2.4 Preparation of the Modified Electrode

The modified electrode was fabricated following the procedure described by Abbas et al.^27^ with minor modifications. Briefly, [Cu­(C_8_H_4_O_4_)]_*n*_ was mixed with graphite powder in varying proportions (10–30% w/w). Mineral oil (20% w/w) was then added, and the mixture was thoroughly ground with a pestle for approximately 20 min until a homogeneous paste was obtained. The resulting paste was packed into a cylindrical plastic tube containing a copper wire to establish electrical contact. Prior to electrochemical measurements, the electrode surface was leveled and polished by gently rubbing it on clean paper to ensure a smooth and reproducible surface. For comparison, an unmodified CPE was prepared using graphite powder and mineral oil in a fixed weight ratio of 80:20 (w/w), which was determined as the optimized proportion.

### 2.5 Sample Preparation

A synthetic urine solution was prepared following the procedure adapted from Meireles et al.^18^ The solution contained CaCl_2_ (0.28 g), KCl (0.40 g), KH_2_PO_4_ (0.35 g), NaCl (0.73 g), NH_4_Cl (0.25 g), and urea (6.25 g) were dissolved in a 250 mL volumetric flask. The volume was adjusted to the mark with 0.1 mol L^–1^ phosphate buffer (pH 6.0), which was also used as the supporting electrolyte. Egg white samples were prepared by diluting raw egg white (1:500, v/v) in 0.1 mol L^–1^ phosphate buffer (pH 6.0). The suspension was homogenized by vigorous stirring for 20 min, following the protocol described by Priya et al.^28^

### 2.6 Electrochemical Measurement

Electrochemical measurements were carried out using a PGSTAT101 potentiostat/galvanostat (Autolab, Metrohm, FL, USA), controlled by NOVA 2.1 software. A standard three-electrode configuration was used, consisting of an Ag/AgCl (3.0 mol L^–1^ KCl) reference electrode, a platinum wire coil as counter electrode, and carbon paste-based working electrodes, either unmodified or modified with [Cu­(C_8_H_4_O_4_)]_*n*_.

Electrochemical characterization of CIP was performed using CV and electrochemical impedance spectroscopy (EIS). CV measurements were conducted within a potential window of 0.0–1.6 V at a scan rate of 50.0 mV s^–1^. The effects of phosphate buffer pH (4.0, 6.0, 7.0, and 8.0) as the supporting electrolyte and varying [Cu­(C_8_H_4_O_4_)]_*n*_ loadings (10%, 20%, and 30%, w/w) in the electrode formulation was systematically investigated.

EIS measurements were carried out using the ferricyanide/ferrocyanide redox couple in 0.1 mol L^–1^ KCl solution containing 5.0 mmol L^–1^ of K_3_Fe­(CN)_6_ and K_4_Fe­(CN)_6_. Impedance spectra were recorded over a frequency range of 100 kHz to 100 mHz using a 10 mV sinusoidal perturbation. All measurements were performed in triplicate (*n* = 3).

### 2.7 Analytical Performance

Prior to constructing the calibration curves, the experimental conditions were optimized using differential pulse voltammetry (DPV) and square wave voltammetry (SWV). To maximize sensitivity, default instrumental parameters were maintained during all measurements. Following optimization, calibration curves for CIP quantification were obtained using DPV under the refined conditions. The main analytical parameters, including the linear dynamic range, sensitivity, limit of detection (LOD), and limit of quantification (LOQ), were determined from the calibration curve. The LOD and LOQ were calculated according to eqs 2 and 3, respectively

2 LOD=3×sbm

3 LOQ=10×sbm

Where “*s*_b_” represents the standard deviation of blank measurements (*n* = 10), while “*m*” is the analytical sensitivity (slope obtained from analytical curve).

To assess the applicability of [Cu­(C_8_H_4_O_4_)]_*n*_/CPE, recovery tests were conducted using synthetic urine and egg samples spiked with CIP at two concentration levels (5.00 and 23.6 μmol L^–1^). The CIP content was quantified in triplicate by measuring the corresponding current responses. Results were reported as recovery percentages and relative standard deviation (RSD), with recoveries calculated according to eq 4

4 $$\mathrm{Recovery}\;(\%)=\frac{C_{\mathrm{final}}}{C_{\mathrm{initial}}}\times 100$$

Where *C*_final_ represent the measured concentration of CIP, and *C*_initial_ corresponds to the spiked concentration.

Additionally, to assess the selectivity of the electrode toward CIP, calibration curves were constructed in the absence and presence of common potential interfering substances, including ascorbic acid, uric acid, urea, glucose, Na^+^, Ca^+^ and enrofloxacin. The influence of these interferents was quantified using eq 5

5 $$\mathrm{Error}\;\mathrm{variance}\;(\%)=\left(\frac{S_{\mathrm{presence}}-S_{\mathrm{absence}}}{S_{\mathrm{absence}}}\right)\times 100$$

Where *S*_presence_ represents the signal of CIP in the presence of interferents, and *S*_absence_ is the signal of CIP in their absence.

## 3 Results and Discussion

### 3.1 Physical and Structural Characterization of [Cu­(C_8_H_4_O_4_)]_*n*_

After synthesis, the material, [Cu­(C_8_H_4_O_4_)]_*n*_, was characterized by FT-IR analysis to confirm its formation and analyze its structural features. The FT-IR spectrum of [Cu­(C_8_H_4_O_4_)]_*n*_ (Figure 1A) exhibits distinct absorption bands within the range of 4000–400 cm^–1^, providing insights into the interactions between the H_2_BDC ligand and the copper metal center. Notably, the bands observed between 500 and 1000 cm^–1^ are attributed to Cu–O stretching and bending vibrations, indicative of metal–ligand coordination. The absorption peaks at 1388, 1499, and 1560 cm^–1^ correspond to C–O stretching vibrations, C=C, and C=O, respectively.^29^ Additional bands at 1018 and 1102 cm^–1^ are associated with C–O and C–C stretching vibrations, respectively.^30^ A broad band observed around 3500 cm^–1^ is linked to surface-adsorbed water and hydroxyl groups within the as-synthesized structure.^31^

**Figure 1:**
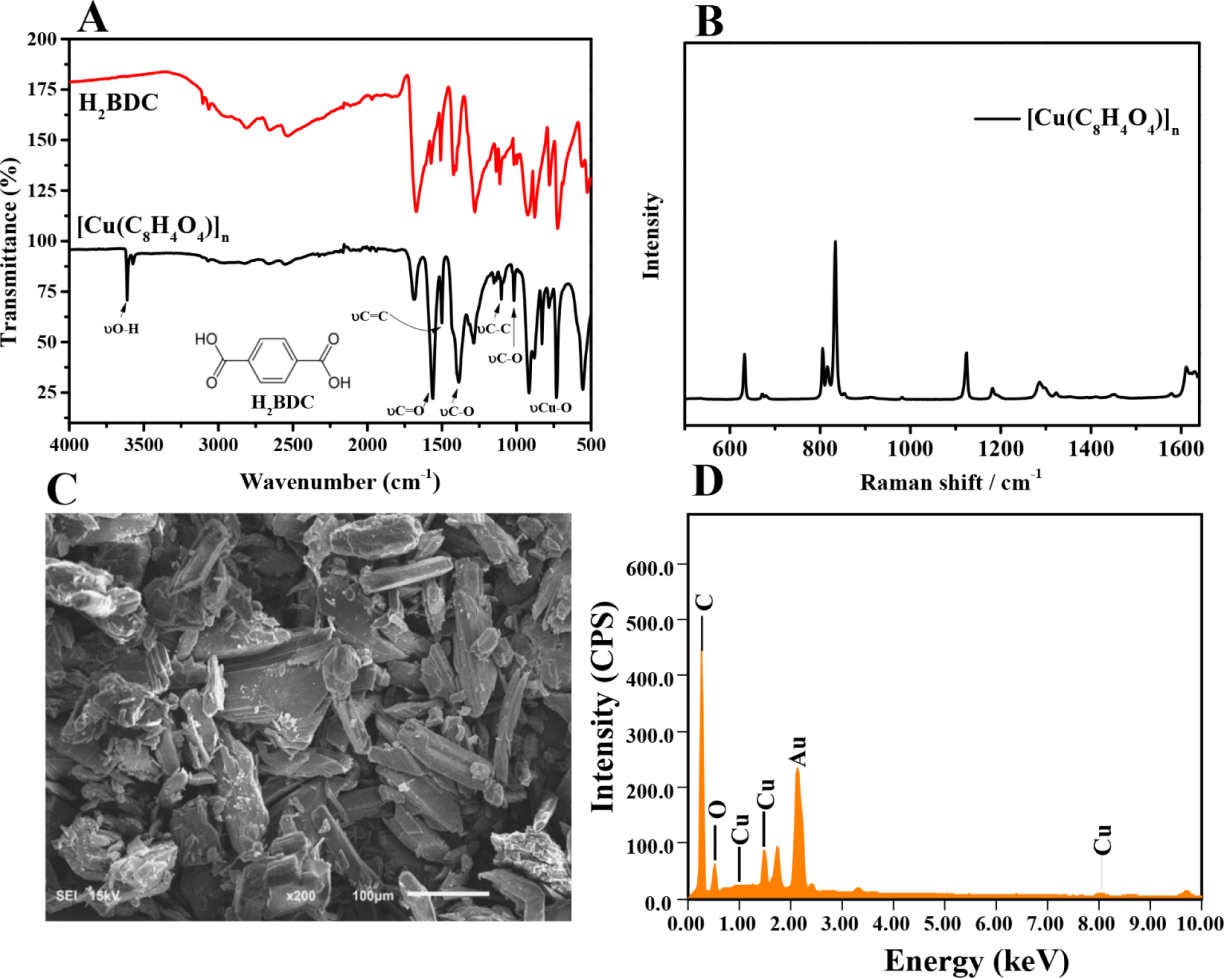
Characterization of [Cu­(C_8_H_4_O_4_)]_*n*_ by (A) Fourier transform infrared (FT-IR); (B) Raman Spectroscopy; (C) Scanning electron microscopy (SEM) and (D) Energy Dispersive Spectroscopy (EDS).

Furthermore, Raman spectroscopy was performed to obtain additional information into the metal–ligand interactions, especially in low-frequency regions, where Cu–O vibrations are more evident.^32^ Thus, in the Raman spectrum of [Cu­(C_8_H_4_O_4_)]_*n*_ (Figure 1B), a series of characteristic bands are observed that reflect the coordination between the metal ion and the H_2_BDC ligand. In the high-frequency region (≥900 cm^–1^), the stretching vibration at 1620 cm^–1^ that is related to C = C of the H_2_BDC aromatic ring, the band at 1530 cm^–1^ that deals with the asymmetric vibration of the COO^–^ group and the bands 1436 and 1419 cm^–1^ referring to the symmetric vibrations of the COO^–^ group stand out. These bands indicate the presence of the H_2_BDC ligand and suggest that the aromatic ring structure is preserved during the formation of the MOF.^33^ In the low-frequency region (100–750 cm^–1^), new bands appear that are attributed to Cu–O vibrations, evidencing the formation of the MOF. Specifically, bands observed below ≤ 800 cm^–1^ are associated with Cu–O vibrational modes, confirming coordination between the Cu^2+^ ion and the H_2_BDC ligand. Furthermore, a band at approximately 600 cm^–1^ may be attributed to other vibrations involving the metal center.^34^

The surface structure and morphology of [Cu­(C_8_H_4_O_4_)]_*n*_ were characterized by SEM, as shown in Figure 1C. The micrograph reveals a heterogeneous surface composed of well-defined, plate-like crystals with regular edges and varying sizes. These particles exhibit a layered or lamellar morphology, typical of some MOFs synthesized under solvothermal conditions. Elemental analysis by EDS (Figure 1D) confirmed the presence of Cu, C, and O, consistent with the formation of the coordination complex and in agreement with previously reported data.^35^

Additionally, XRD analysis (Figure 2A) was conducted to evaluate the crystallinity and phase composition of [Cu­(C_8_H_4_O_4_)]_*n*_. The diffraction pattern exhibits sharp and well-defined peaks, indicative of high crystallinity, with prominent reflections at 2θ values of approximately 18°, 25°, and 30°. These results are consistent with those reported by Al-Harbi et al.^36^ Furthermore, comparison with the crystallographic pattern of ICSD 1381 (Cu_16_O_14_) (Figure 2A, inset) shows a strong similarity, confirming the crystalline structure of the synthesized material. The presence of these well-defined diffraction peaks confirms the successful formation of the MOF and suggests a well-ordered crystal lattice. This structural integrity is essential for the stability and performance of the material.

**Figure 2:**
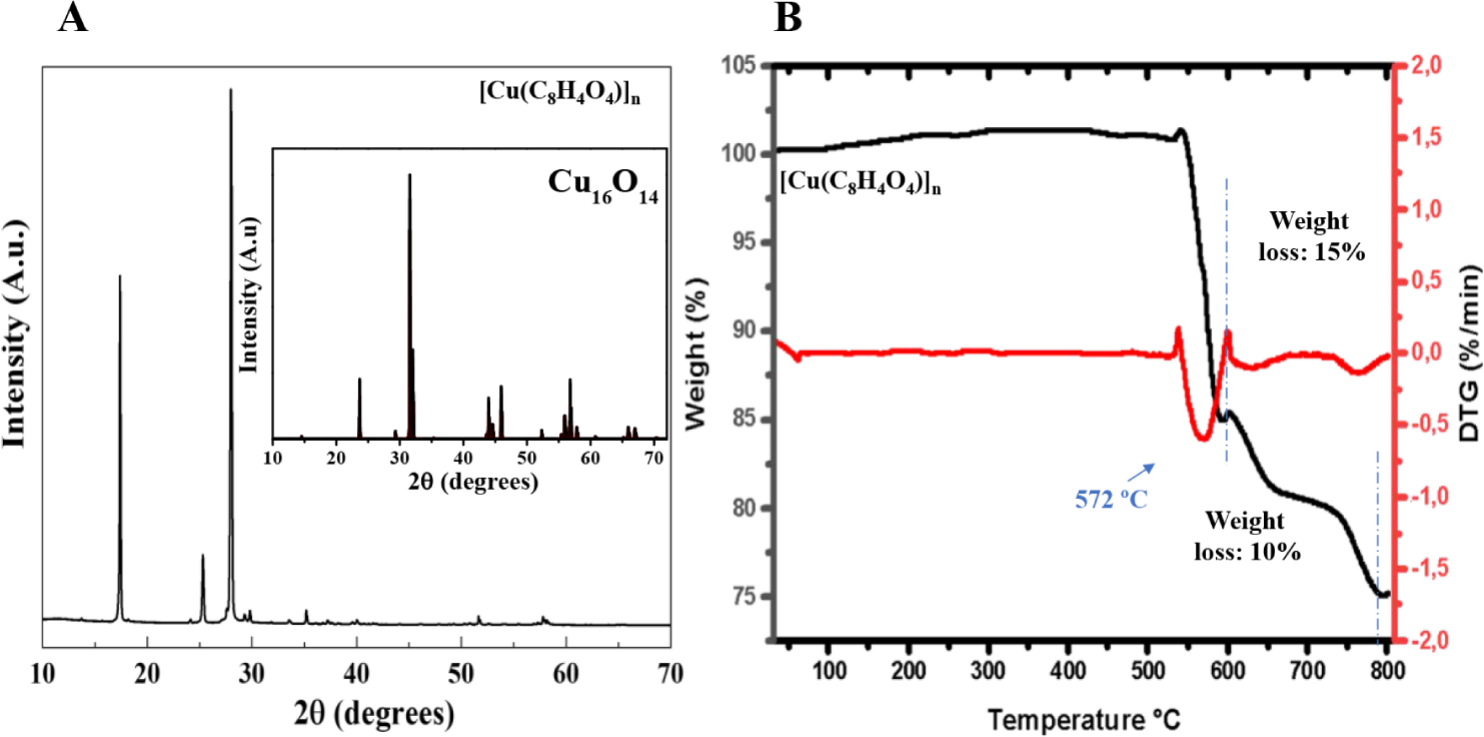
(A) X-ray diffractogram of [Cu­(C_8_H_4_O_4_)]_*n*_; Inset: Crystallographic pattern of ICSD 1381 (Cu_16_O_14_), showing similarity to the experimental data for [Cu­(C_8_H_4_O_4_)]_*n*_ and suggesting possible crystallographic planes in the synthesized material. (B) Thermogravimetric analysis (TGA).

The thermal stability of [Cu­(C_8_H_4_O_4_)]_*n*_ was evaluated by TGA. The TGA curve (Figure 2B) shows a gradual weight loss at lower temperatures, attributed to the evaporation of solvent molecules and the removal of adsorbed functional groups within the framework. A significant weight loss (∼15%) occurs around 572 °C, indicating the onset of structural decomposition. A second pronounced weight loss (∼10%) is observed near 600 °C, corresponding to the degradation of the organic ligand and the collapse of the MOF framework. These results demonstrate that [Cu­(C_8_H_4_O_4_)]_*n*_ exhibits high thermal stability, withstanding elevated temperatures prior to substantial structural breakdown.

In the context of electrochemical sensor development, this result is particularly relevant since the applied conditions involve potential within the aqueous window (e.g., 0.6 to +1.2 V vs Ag/AgCl) at room temperature, which is considerably milder than the thermal stress that leads to decomposition above 500 °C. Therefore, the thermal stability evidenced by TGA strongly reinforces the idea that the MOF structure can remain structurally stable under the electrochemical conditions employed for sensor development.^37^

BET analysis revealed a specific surface area of 6.047 m^2^ g^–1^, a total pore volume of 0.0131 cm^3^ g^–1^, and an average pore diameter of 34.726 nm, indicating a predominantly mesoporous structure. The nitrogen adsorption–desorption isotherms (Figure 3) exhibited a type III profile with an H3 hysteresis loop. These findings highlight key features of the material, emphasizing its combined mesoporosity and microporosity, advantageous for modifying electrochemical sensors.

**Figure 3:**
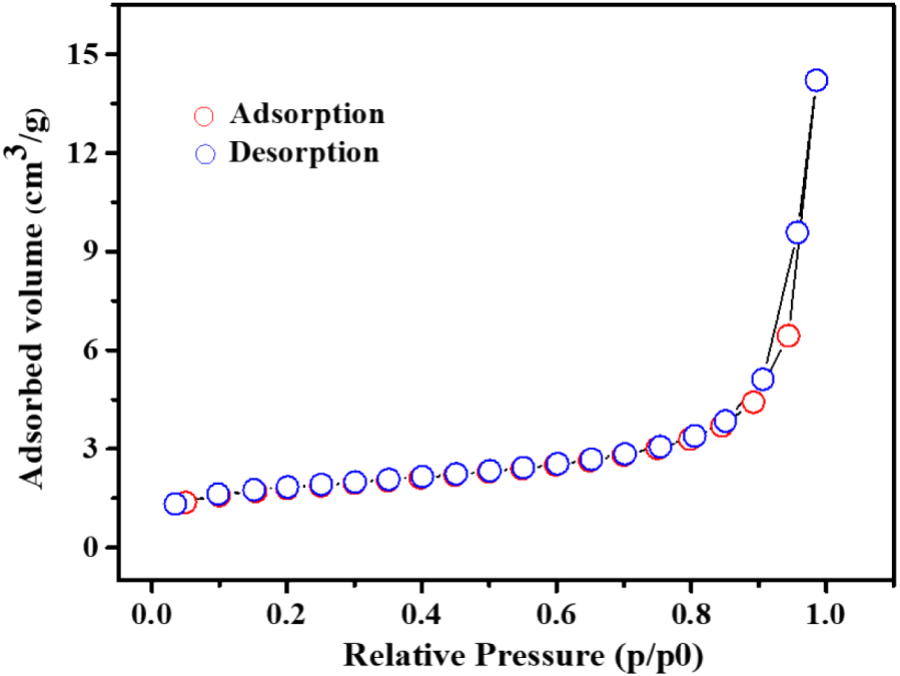
Characterization of [Cu­(C_8_H_4_O_4_)]_*n*_ by nitrogen adsorption–desorption isotherm.

### 3.2 Electrochemical Characterization of [Cu­(C_8_H_4_O_4_)]_*n*_/CPE

To evaluate the electrochemical properties of the [Cu­(C_8_H_4_O_4_)]_*n*_ modified electrode, CV and EIS were conducted using the ferricyanide/ferrocyanide redox couple ([Fe­(CN)_6_]^3–^/^4–^) as a probe. Figure 4A presents the cyclic voltammograms of the unmodified CPE and the [Cu­(C_8_H_4_O_4_)]_*n*_/CPE. The CV data revealed that the [Cu­(C_8_H_4_O_4_)]_*n*_ modified electrode exhibited higher peak currents compared to the unmodified electrode, confirming enhanced electrochemical activity. Furthermore, the peak-to-peak potential separation (Δ*E*_p_ = *E*_pa_ – *E*_pc_), where *E*_pa_ and *E*_pc_ are the anodic and cathodic peak potentials, respectively, decreased from 349.13 mV for the unmodified CPE to 161.14 mV for the [Cu­(C_8_H_4_O_4_)]_*n*_/CPE. This reduction indicates accelerated electron transfer kinetics and improved reversibility of the redox process at the modified electrode.

**Figure 4:**
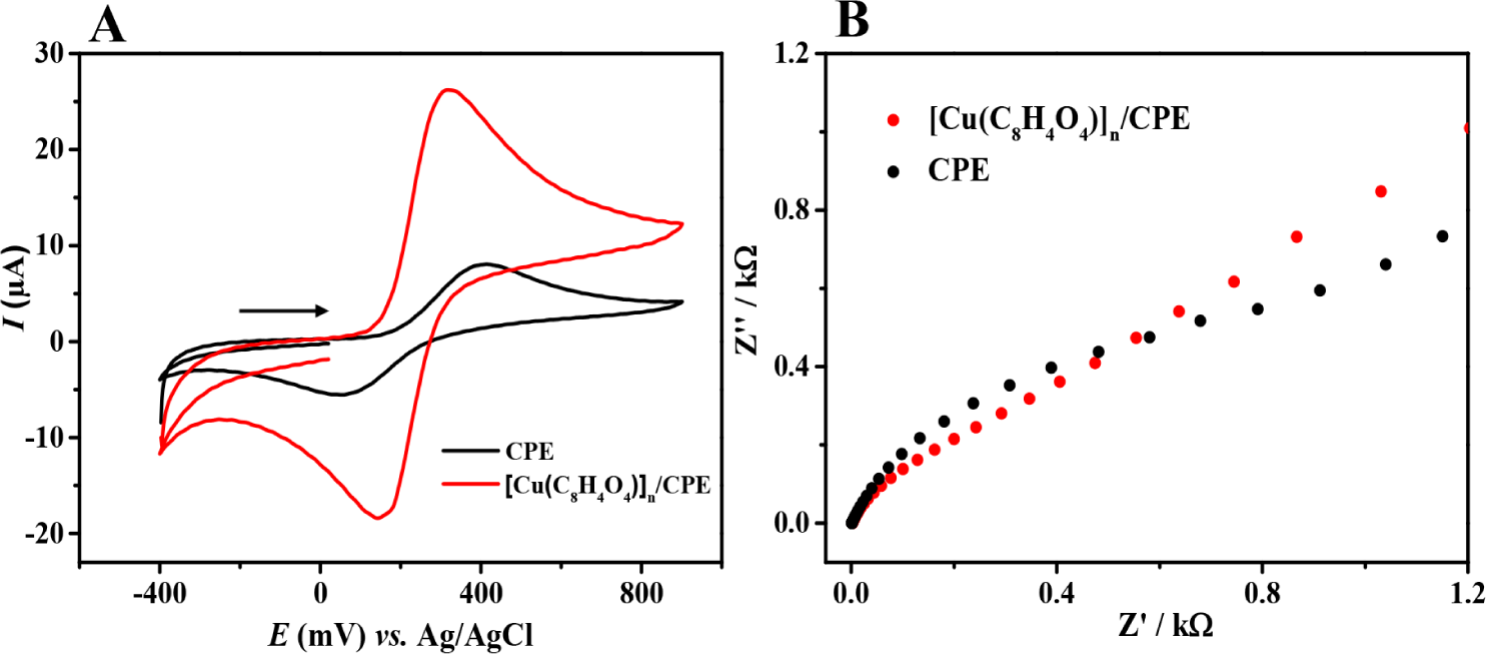
(A) Cyclic voltammograms (scan rate of 50 mV s^–1^) and (B) Nyquist diagrams recorded using bare CPE and [Cu­(C_8_H_4_O_4_)]_*n*_/CPE in 0.1 mol L^–1^ KCl solution containing 1.0 mmol L^–1^ K_3_Fe­(CN)_6_ and K_4_Fe­(CN)_6_.

The Nyquist plots presented in Figure 4B were obtained under identical experimental conditions for both the unmodified CPE and for [Cu­(C_8_H_4_O_4_)]_*n*_/CPE. In these plots, the diameter of the semicircular arc in the low-frequency region corresponds to the charge transfer resistance (*R*_ct_), which reflects the facility of electron transfer at the electrode–electrolyte interface. A smaller semicircle diameter is indicative of a lower *R*_ct_, suggesting a more efficient electron transfer. For the unmodified CPE, the *R*_ct_ was approximately 116.6 Ω, while for the [Cu­(C_8_H_4_O_4_)]_*n*_/CPE, it decreased to about 73.8 Ω. This significant reduction in *R*_ct_ after modification with [Cu­(C_8_H_4_O_4_)]_*n*_ confirms the enhanced electrochemical performance of the [Cu­(C_8_H_4_O_4_)]_*n*_/CPE, consistent with the results from the CV analyses.

To better assess the kinetics of the electron transfer process, the heterogeneous electron transfer rate constant (*k*_0_) was calculated using eq 6:

6 $$k_{0}=\frac{RT}{nF}\times \frac{1}{R_{ct}}$$

where *R* is the universal gas constant (8.314 J mol^–1^ K^–1^), T is the absolute temperature (K), n is the number of electrons participating in the redox reaction (typically 1 for the pair [Fe­(CN)_6_]^3–^/^4–^), and F is Faraday’s constant (96.485 C mol^–1^). At room temperature (298 K), this equation enables the quantitative evaluation of the electron-transfer rate at the electrode–electrolyte interface.

The calculated *k*_0_ values for the unmodified and [Cu­(C_8_H_4_O_4_)]_*n*_/CPE electrodes were 2.2 × 10^–4^ cm s^–1^ and 3.5 × 10^–4^ cm s^–1^, respectively, further confirming the enhanced electrochemical activity imparted by [Cu­(C_8_H_4_O_4_)]_*n*_ modification. These results demonstrate the effectiveness of [Cu­(C_8_H_4_O_4_)]_*n*_ as a functional modifier to improve the electron-transfer characteristics of the electrode.

In addition to charge-transfer analysis, the electroactive surface area was estimated by CV using the Randles–Ševčík equation with [Fe­(CN)_6_]^3–^/^4–^ as the redox probe. The bare CPE exhibited an electroactive surface area of 0.021 cm^2^, whereas the [Cu­(C_8_H_4_O_4_]_*n*_/CPE reached 0.084 cm^2^, representing a 4-fold increase. This substantial increase in electroactive surface area indicates a higher density of accessible active sites on the modified surface and, together with the lower *R*_ct_ and higher *k*_0_ values obtained, corroborates the improved electron-transfer kinetics and overall electrochemical performance of the [Cu­(C_8_H_4_O_4_)]_*n*_-modified electrode.

The electrochemical behavior of the Fe­(CN)_6_^3–/4–^ redox couple at the [Cu­(C_8_H_4_O_4_)]_*n*_/CPE interface was further investigated by evaluating the effect of different scan rates (ν). Figure 5A shows cyclic voltammograms of [Cu­(C_8_H_4_O_4_)]_n_/CPE recorded at scan rates ranging from 10 to 200 mV s^–1^. The anodic peak currents (*I*_pa_) showed a linear increase with the square root of the scan rate (ν), consistent with the Randles–Sevcik equation, which describes diffusion-controlled reversible redox process. A plot of log *I*_pa_ versus log ν was constructed (Figure S1A), yielding a strong linear correlation expressed by the regression equation log (*I*_pa_) = 0.43009 log *v* + 0.0584, *R* ^2^ = 0.9985.

**Figure 5:**
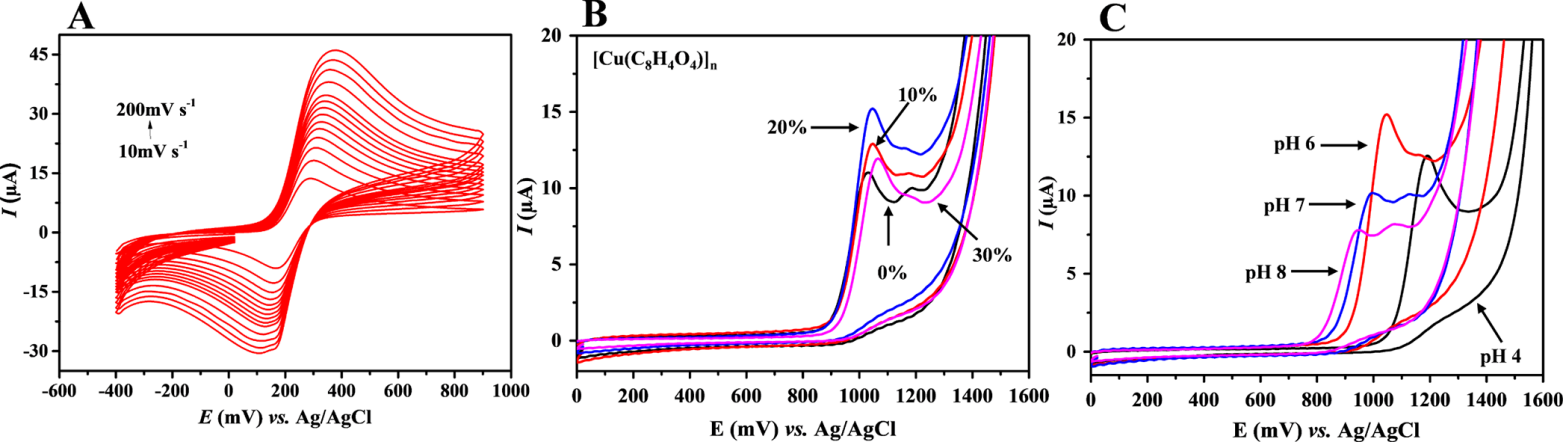
(A) Cyclic voltammograms of Fe­(CN)­6^3–/4–^ at different scan rates (10–200 mV s^–1^). All measurements were performed using the 20%-[Cu­(C_8_H_4_O_4_)]_*n*_/CPE electrode in 0.1 mol L^–1^ phosphate buffer solution (pH 6) containing 5.0 × 10^–4^ mol L^–1^ of Fe­(CN)­6^3–/4–^. (B) Voltammograms of ciprofloxacin (CIP) (44.3 μmol L^–1^) using unmodified and modified electrode with different percentages of the [Cu­(C_8_H_4_O_4_)]_*n*_ (10, 20, and 30%, w/w) in a phosphate buffer (0.1 mol L^–1^, pH 6) at a scan rate of 50 mV s^–1^. (C) Cyclic voltammograms recorded using the 20%-[Cu­(C_8_H_4_O_4_)]_*n*_/CPE electrode for 44.3 μmol L^–1^ of CIP solution prepared in 0.1 mol L^–1^ phosphate buffer at different pHs and *v* = 50 mV s^–1^.

This high correlation coefficient (*R* ^2^ = 0.9985) confirms an excellent fit with the expected behavior for a reversible redox process. The slope of 0.43009 suggests that the electrochemical process is predominantly diffusion-controlled, with possible contribution from adsorption phenomena, as the value is close to the theoretical slope of 0.5 for a purely diffusion-controlled mechanism. These results highlight the improved electrochemical behavior of the [Cu­(C_8_H_4_O_4_)]_*n*_-modified electrode, demonstrating its suitability for applications requiring efficient electron transfer and high sensitivity.^38^ Furthermore, the linear dependence of peak current on the square root of the scan rate (*I*_p_ versus ν^1/2^), shown in Figure S1B, provided additional confirmation of a diffusion-driven mechanism.

### 3.3 Electrochemical Behavior of Ciprofloxacin

The electrochemical detection of CIP at the modified electrode surface can be influenced by several factors, including (i) the concentration of the modifying agent, (ii) the pH of the electrolyte solution, and (iii) the voltametric technique employed. Optimizing these parameters is essential for enhancing the overall performance of the sensor. The following section details the optimization procedures carried out for the [Cu­(C_8_H_4_O_4_)]_*n*_-modified electrode.

#### 3.3.1 Effect of Modifying Agent Concentration

The electrochemical behavior of CIP was initially investigated using electrode modified with different loading of [Cu­(C_8_H_4_O_4_)]_*n*_ (10%, 20%, and 30% by mass). The experiments were carried out in a 44.3 μmol L^–1^ CIP solution prepared in phosphate buffer (pH 6.0) at a scan rate of 50 mV s^–1^. Figure 5B presents the cyclic voltammograms of the unmodified CPE and the modified electrodes at varying [Cu­(C_8_H_4_O_4_)]_*n*_ concentrations, all in the presence of CIP. In all cases, the appearance of a distinct oxidation peak confirmed the electroactive nature of CIP. A single anodic peak was observed in all modified electrodes, indicating the irreversible oxidation of CIP. In addition, the anodic current response increased with higher [Cu­(C_8_H_4_O_4_)]_*n*_ loadings, demonstrating that the modification enhances electrode conductivity and promotes more efficient electron transfer, thereby facilitating CIP oxidation. This observation is consistent with previous reports in which MOFs were employed as electrode modifiers, leading to improved electrochemical performance.^39^

The incorporation of [Cu­(C_8_H_4_O_4_)]_*n*_ into the electrode surface significantly increased the oxidation current compared to the unmodified electrode, indicating improved adsorption and higher detection sensitivity for CIP. Interestingly, this effect was most pronounced for the electrode modified with 20% [Cu­(C_8_H_4_O_4_)]_*n*_ (Figure S2). We hypothesize that a 20% loading achieved a balance between the number of electroactive sites and the conductivity of the carbon paste. At 10% loading, the current response was superior to that of bare CPE, but the limited density of active sites likely limited overall electrochemical performance. In contrast, a 30% loading resulted in a slight decrease in current, likely due to partial obstruction of the carbon conductive network, which hindered electron transfer. Therefore, the 20% [Cu­(C_8_H_4_O_4_)]_*n*_ content produced the best electrochemical response, effectively maximizing current while maintaining sufficient conductivity. This enhancement can be attributed to the increased surface area and improved conductivity provided by the [Cu­(C_8_H_4_O_4_)]_*n*_ modification, which facilitates electron transfer and introduces additional active sites for CIP interaction. These findings are consistent with previous reports, where electrode modifications were shown to enhance electrochemical performance. For instance, the incorporation of graphene nanosheets and Fe_3_O_4_ nanoparticles as modifiers significantly improved the electrochemical detection of CIP by promoting faster electron transfer and increasing the availability of adsorption sites.^39^ These results highlight the effectiveness of [Cu­(C_8_H_4_O_4_)]_*n*_ as a modifier for enhancing the electrochemical detection of CIP.

#### 3.3.2 Effect of pH

The pH of the supporting electrolyte plays a crucial role in modulating the electrochemical behavior of CIP on the [Cu­(C_8_H_4_O_4_)]_*n*_ modified electrode. To determine the optimal pH for CIP detection, CV experiments were conducted using 0.1 mol L^–1^ phosphate buffer solutions with pH values ranging from 4.0 to 8.0 (Figure 5C). The results indicated that acidic conditions enhanced the anodic CIP signal, with the highest current peak observed at pH 6.0 (Figure S3A). This enhancement suggests that protonation of CIP facilitates its electrochemical oxidation, since the drug exists in its cationic form (CIP^+^) under acidic conditions, which is more easily oxidized compared to its neutral or anionic forms.^40^

The observed optimal pH can be rationalized based on the acid–base properties of CIP, which contains a carboxylic group with a p*K*_a_ of approximately 6.1 and a piperazine moiety with a p*K*_a_ of approximately 8.7. At pH 6.0, CIP predominantly exists as a zwitterion, featuring a deprotonated carboxyl group and a protonated piperazine nitrogen. This protonation state enhances electrostatic interactions with negatively charged sites on the [Cu­(C_8_H_4_O_4_)]_*n*_ surface, facilitating adsorption and promoting efficient electron transfer, thereby generating the higher anodic current observed. At pH values below or above 6.0, these interactions are decreased due to changes in CIP speciation, resulting in reduced analytical signals.^41^ Therefore, a phosphate buffer solution at pH 6.0 was selected as the most suitable electrolyte for subsequent experiments.

The analysis of mass transport at the modified electrode revealed a clear dependence of the peak potential (*E*_p_) on the solution pH (Figure S3B), exhibiting a linear relationship as expressed in eq 7:

7 $$E_{p}\left(V\right)=1.144-0.0646pH\left(R^{2}=0.992\right)$$

This linear behavior is consistent with the Laviron eq (eq 8), which allows the determination of the number of electrons involved in the electrochemical process:

8 $$E_{pa}=E^{0}+\left(RT/\alpha nF\right)\ln\left(RTk^{0}/\alpha nF\right)+\left(RT/\alpha nF\right)\ln\nu$$

In this equation, *k* ^0^ represents the standard rate constant of the reaction, while α is the electron transfer coefficient, providing insights into the reaction’s symmetry and kinetics. Specifically, α indicates the fraction of the electron transfer process that controls the rate-determining step.

The electrochemical data obtained during the reaction allowed the calculation of the number of electrons involved. From the slope of the linear fit, the number of electrons was determined to be 1.92 (α = 0.06), indicating that the electrochemical oxidation of the analyte follows a two-electron transfer mechanism. These results are consistent with previous studies in which the electrochemical oxidation of CIP was investigated using various electrode modifications. For instance, the oxidation of CIP on the surface of graphene-modified electrodes was considered an irreversible process, and the number of electrons involved was determined to be two based on the application of the Laviron equation.^42^

#### 3.3.3 Pulsed Voltammetric Technique

A comparative evaluation between DPV and SWV was performed to determine the most suitable technique for the electrochemical detection of CIP under identical experimental conditions. Figure 6A presents the results. As observed in the figure, DPV exhibited a significantly higher current response compared to SWV, indicating superior analytical sensitivity for CIP. Thus, DPV was selected for subsequent methodological development.

**Figure 6:**
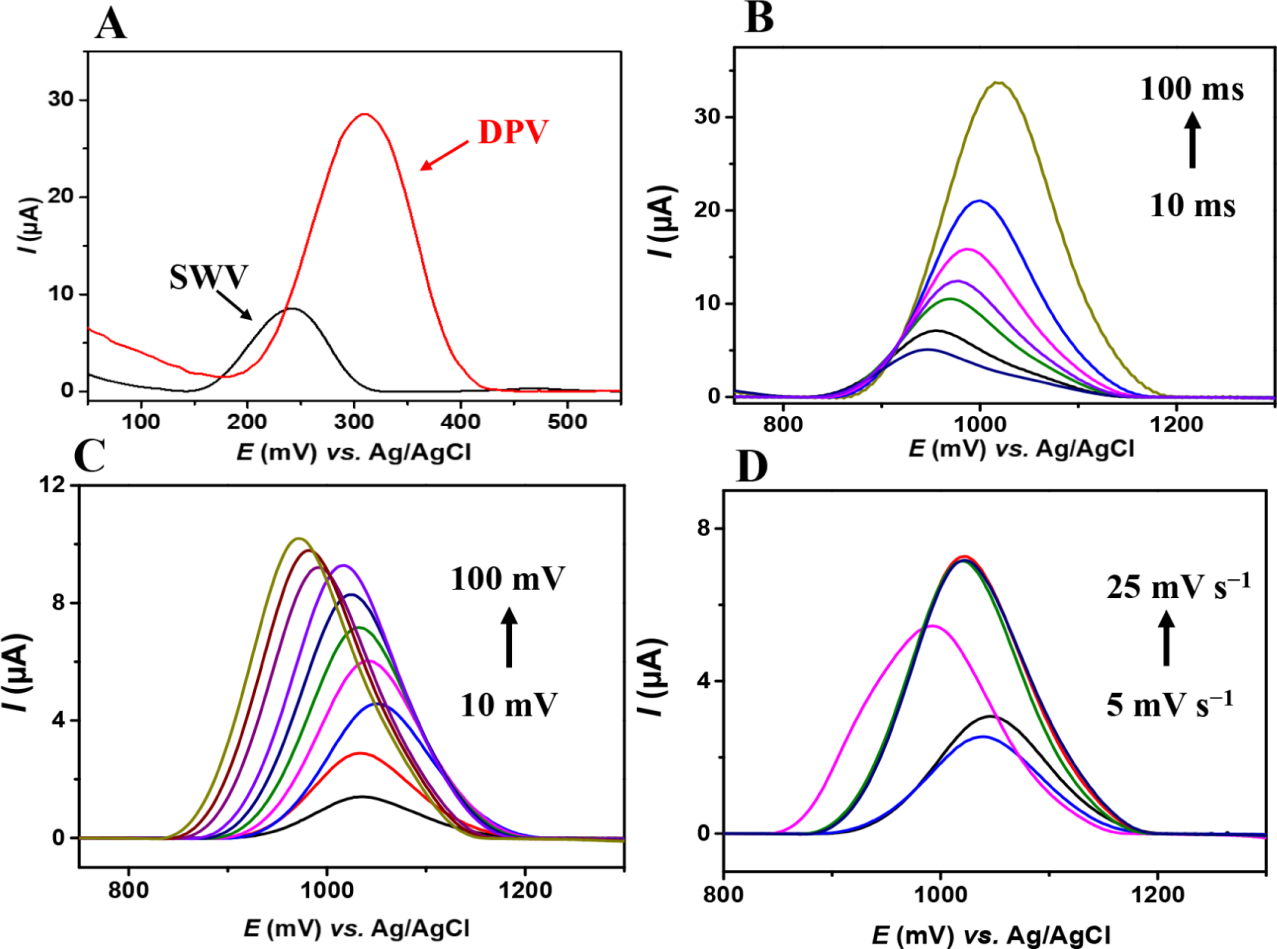
(A) Comparison between differential pulse voltammetry (DPV) and square wave voltammetry (SWV) techniques for ciprofloxacin (CIP) detection; DPV curves recorded for the optimization of (B) modulation time, (C) pulse amplitude, and (D) scan rate using the 20%-[Cu­(C_8_H_4_O_4_)]_*n*_/CPE electrode in a 44.3 μmol L^–1^ CIP solution prepared in 0.1 mol L^–1^ phosphate buffer (pH 6).

To further improve the analytical performance of the technique, important instrumental parameters were optimized, including (i) modulation time (10–100 ms, Figure 6B), (ii) pulse amplitude (10–100 mV, Figure 6C), and (iii) scan rate (5, 10, 15, 20, and 25 mV s^–1^, Figure 6D). The best results were obtained using the optimal conditions of (i) 100 ms, as modulation time, (ii) 100 mV pulse amplitude, and (iii) 25 mV s^–1^ scan rate. These parameters were applied consistently throughout the study to ensure reproducible signals and robust analytical performance.

Previous studies indicate that DPV offers advantages in terms of sensitivity and selectivity for CIP detection, resulting in lower LOD compared to SWV. For instance, a sensor based on copper–iron mixed oxides and reduced graphene (CIMMO/rGO/GCE) showed an LOD of 0.47 nmol L^–1^ for CIP using DPV, while SWV showed an LOD of 0.92 mmol L^–1^, indicating lower sensitivity than DPV.^43^ Furthermore, DPV is recognized for its ability to minimize background current, thereby enhancing the signal-to-noise ratio, which is particularly advantageous for detecting low analyte concentrations in complex matrices.

### 3.4 Evaluation of Analytical Performance

Following the optimization of experimental parameters, the analytical performance of the proposed method was evaluated in three different matrices: (i) phosphate buffer solution, (ii) synthetic urine, and (iii) egg white. The method demonstrated a wide linear detection range, specifically from 2.15 to 20.2 μmol L^–1^ in phosphate buffer, and from 2.52 to 23.6 μmol L^–1^ in both synthetic urine and egg white. Calibration curves for each matrix are presented in Figures S4A, B and C, corresponding to phosphate buffer, synthetic urine, and egg white, respectively. All calibration curves exhibited excellent linearity, with determination coefficients (*R* ^2^) greater than 0.99.

The method demonstrated significant sensitivity, reaching an LOD of 0.5 μmol L^–1^ and a LOQ of 1.7 μmol L^–1^ in synthetic urine. For egg white, the LOD and LOQ were 3.0 μmol L^–1^ and 10.1 μmol L^–1^, respectively. Furthermore, the accuracy of the method was evaluated through recovery experiments in synthetic urine and egg samples, spiked with CIP at two concentration levels (5.00 and 23.60 μmol L^–1^). As detailed in Table 1, recovery value ranged from 78.76% to 108.30%, confirming the satisfactory performance of the modified electrode for CIP detection in complex matrices.

**Table 1:** Determination of CIP in Synthetic Urine and Egg White Samples Using the Modified Electrode ([Cu­(C_8_H_4_O_4_)]_*n*_/CEP)

	CIP Concentration (μmol L^–1^)		
Samples	Added	Found	Recovery (±RSD^a^%)
Urine	5.00	5.42 ± 0.14	108.30 ± 2.58
	23.60	22.00 ± 1.06	93.22 ± 4.81
Egg white	5.00	3.94 ± 0.32	78.76 ± 8.12
	23.60	24.30 ± 2.47	102.96 ± 10.16

a Relative Standard Deviation (*n* = 3).

Recovery results for CIP in egg white samples exhibited greater variability compared to synthetic urine, as reflected by larger RSDs and deviations from nominal concentrations. At the lowest concentration level (5.00 μmol L^–1^), recovery was 78.76 ± 8.12%, while at the highest concentration (23.60 μmol L^–1^), it reached 102.96 ± 10.16%. This variability can be attributed to the complex and heterogeneous nature of the egg white matrix, which contains proteins, lipids, and other macromolecules that can interact with CIP or the electrode surface, potentially hindering analyte diffusion and adsorption.^44^ Factors such as protein binding, viscosity, and sample microheterogeneity likely contribute to signal suppression or enhancement, resulting in higher RSDs compared to the more homogeneous synthetic urine.^44^ However, these results demonstrate that the developed sensor effectively detects the target analyte over a wide concentration range while maintaining satisfactory accuracy and precision. Furthermore, the sensor exhibited good repeatability, as indicated by relatively low RSD values (<10.16%, *n* = 3), ensuring reliable performance for various analytical applications.

### 3.5 Effect of Interference on Electrochemical Detection

The anti-interference performance of the [Cu­(C_8_H_4_O_4_)]_*n*_/CEP sensor was assessed by recording DPV curves for CIP in the absence and presence of potential interferents, including ascorbic acid, uric acid, urea, glucose, Ca^+^, Na^+^ and enrofloxacin. The interference effects were quantified by comparing the peak currents obtained with and without each interferent at two analyte-to-interferent ratios (1:1 and 1:5), and the corresponding error values are summarized in Table 2.

**Table 2:** Effect of Different Interfering Species on the Detection of CIP in Synthetic Urine and Egg White Samples Using [Cu­(C_8_H_4_O_4_)]_*n*_/CPE

Interferences	Concentration ratios	Sample urine error variation (%)	Sample egg white error variation (%)
Urea	1:1	+4.43	+3.31
	1:5	+4.27	– 8.19
Ascorbic acid	1:1	–10.31	+9.84
	1:5	–13.50	–10.67
Uric acid	1:1	–13.42	–1.62
	1:5	–15.31	–10.83
Glucose	1:1	–13.14	–4.84
	1:5	–8.82	–2.44
Na^+^	1:1	–7.97	+9.66
	1:5	+3.99	–1.88
Ca^+^	1:1	+2.21	–5.62
	1:5	+5.26	–14.44
Enrofloxacin	1:1	+1.45	–5.07
	1:5	+23.25	–27.28

As shown in Table 2, most common interferents, including urea, ascorbic acid, uric acid, glucose, and inorganic ions (Na^+^ and Ca^2+^), caused only moderate signal deviations, with errors generally within ±15% across both urine and egg white matrices, even at a 5-fold excess. These results confirm that the sensor maintains acceptable accuracy under real sample conditions.

In contrast, enrofloxacin, which shares structural similarity and electrochemical behavior with CIP, produced the most pronounced interference. At a 1:1 ratio, the error remained low (+1.45% in urine and −5.07% in egg white), indicating that the sensor can differentiate between the two FQs under equimolar conditions. However, at a 1:5 ratio, the effect became significant, with errors of +23.25% in urine and −27.28% in egg white. These findings demonstrate that while the sensor exhibits satisfactory selectivity for CIP in the presence of enrofloxacin at comparable concentrations, its accuracy is compromised when enrofloxacin is present in substantial excess.

Overall, the data suggest that the [Cu­(C_8_H_4_O_4_)]_*n*_/CPE sensor provides reliable selectivity for CIP determination in complex biological matrices, with only limited susceptibility to structurally related interferents at high concentration ratios. This indicates its practical utility for real-sample analysis, provided there are no extreme concentration imbalances between CIP and enrofloxacin.

### 3.6 Proposed Reaction Mechanisms

The electrochemical detection of CIP using [Cu­(C_8_H_4_O_4_)]_*n*_/CPE is based on a synergistic redox mechanism, in which the reduction of Cu^2+^ to metallic Cu occurs concomitantly with the irreversible oxidation of CIP at the electrode–solution interface.^45^ The Cu-based coordination polymer exhibits a high surface area and porosity, as evidenced by BET analysis, which significantly increases the adsorption capacity of CIP on the sensor surface.^46^ In addition, [Cu­(C_8_H_4_O_4_)]_*n*_ acts as an efficient redox mediator, facilitating electron transfer between CIP and the electrode. The combination of these properties enables the sensitive detection of CIP.

During detection, CIP (a fluoroquinolone characterized by a structure containing conjugated aromatic rings and a piperazine group) is adsorbed onto the surface of the modified electrode and undergoes an irreversible oxidation process, which is controlled by diffusion. This process involves the transfer of two electrons and two protons, leading to the formation of a stable oxidized product (Figure 7).^47^

**Figure 7:**
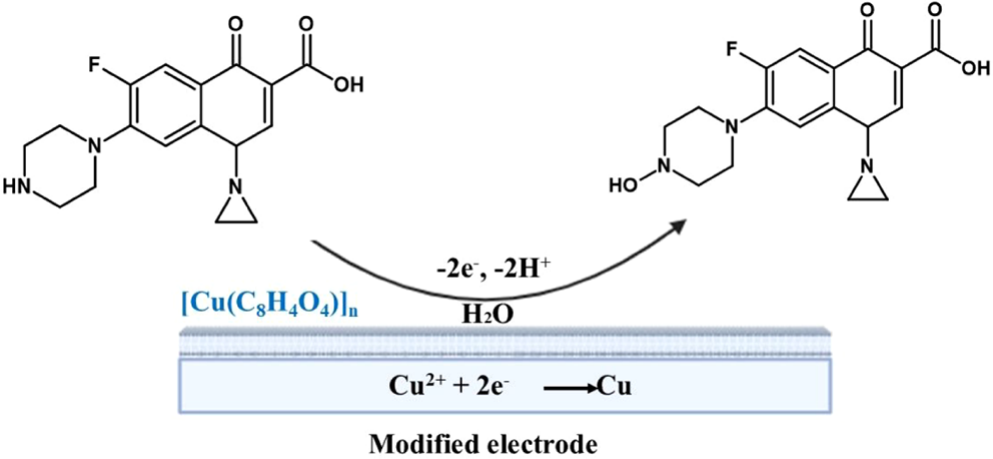
Proposed electrochemical oxidation and reduction mechanism of ciprofloxacin (CIP) on the [Cu­(C_8_H_4_O_4_)]_*n*_ modified electrode surface, illustrating the interaction between CIP and the active sites of the material.

As reported by Rani et al.,^48^ the presence of the Cu^2+^-based MOF facilitates the redox process, acting not only as an adsorbent support but also as an electroactive catalyst. During CIP oxidation, a fraction of Cu^2+^ is reduced to Cu^+^ or Cu^0^ enabling efficient electron transfer. This reversible redox interconversion of Cu, combined with the enhanced electron density at the electrode’s active surface, accounts for the excellent sensitivity and selectivity achieved in the electrochemical detection of CIP.

Figure S5 shows the FT-IR spectra of [Cu­(C_8_H_4_O_4_)]_*n*_ before and after electrochemical detection of CIP as [Cu­(C_8_H_4_O_4_)]_*n*_/CEP. As observed, the band at ∼500 cm^–1^, attributed to the Cu–O stretching vibration, decreases in intensity after the electrochemical process, suggesting partial involvement of Cu centers in the redox mechanism. Furthermore, the appearance of intense absorption bands near 2800 cm^–1^, characteristic of C–H stretching vibrations, indicates the adsorption of CIP on the electrode surface. Together, these spectral changes may support the proposed mechanism involving Cu^2+^/Cu° redox activity in [Cu­(C_8_H_4_O_4_)]_*n*_/CEP and the interaction of CIP during the electrochemical detection process.

### 3.7 Comparative Electrochemical Performance of Modified Electrodes

The [Cu­(C_8_H_4_O_4_)]_*n*_-based electrochemical sensor demonstrated satisfactory sensitivity and detection for CIP, as evidenced by the comparative analysis of the LOD and LOQ values presented in Table 3.

**Table 3:** Comparative Electrochemical Evaluation of Modified Electrodes for CIP Determination^a^

Modified electrode	Sample	Linear range (μmol L^–1^)	LOD (μmol L^–1^)	LOQ (μmol L^–1^)	Technique	Ref.
[Cu(C_8_H_4_O_4_)]_*n*_/CPE	Urine and egg white	2.52–23.6	0.5–3.0	1.7–10.1	DPV	This work
PGE	Pharmaceutical formulations and urine	14.75 −47.5	1.53	4.54	SWV	^49^
CZF/CPE	Urine and serum	0.909 - 4.70 × 10^3^	2.58 × 10^- 3^	NS	ASV	^50^
TiO_2_/PB/AuNPs/CMK-3/Nafion/GE	Environmental water	1–10 and10–52	1.08 × 10^–1^	NS	CV	^51^
*p*(2TM)/GC electrode	Urine	0.1–200	7 × 10 ^- 3^	NS	SWSV	^52^
BIA-AMP and CE-C^4^D	Milk	1–100 and 50–250	3 × 10 ^- 1^-5.0	NS	Amperometry	^53^
MWCNTs-GC	Urine and serum	40–1000	6.0	NS	Amperometry	^54^

a ASV: anodic stripping voltammetry; BIA-AMP: electrode batch-injection analysis with amperometric detection; CE-C4D: capillary electrophoresis with capacitively coupled contactless conductivity detection; CV: cyclic voltammetry; CZF-CME: copper zinc ferrite nanoparticle modified carbon paste electrode; DPV: differential pulse voltammetry; MWCNT/GCE: multiwall carbon nanotubes film-modified glassy carbon electrode; NS: not specified; p­(2TM)/GC electrode: 2-(hydroxymethyl)­thiophene on glassy carbon; PGE: pencil graphite electrode; SWSV: square wave stripping voltammetry; SWV: square wave voltammetry; TiO2/PB/AuNPs/CMK-3/Nafion/GE: graphite electrode modified with titanium dioxide sol, gold nanoparticles and CMK-3 type mesoporous carbon.

From Table 3, we observed that the [Cu­(C_8_H_4_O_4_)]_*n*_/CPE sensor demonstrated competitive analytical performance, with LODs between 0.5 and 3.0 μmol L^–1^ and broad applicability in complex matrices such as urine and egg white. In comparison, PGE^49^ showed higher LOD (1.53 μmol L^–1^) and a restricted linear range, evidencing the greater sensitivity of the MOF-based sensor. Although sensors such as CZF/CPE^50^ and p­(2TM)/GC^52^ achieved low LODs, below 10^–2^ μmol L^–1^, these systems require more elaborate preparation steps and the use of specific modifiers, which can limit reproducibility and practical application in routine analysis. Similarly, complex architectures such as TiO_2_/PB/AuNPs/CMK-3/Nafion/GE^51^ offer good electrochemical response, but involve multiple nanomaterials, increasing costs and limiting scalability. In this context, [Cu­(C_8_H_4_O_4_)]_*n*_ is highlighted because combining simplicity of preparation, structural stability, and good sensitivity, thereby representing as a viable and practical alternative to other electrochemical sensors described in the literature.

Based on these advantages, it is important to highlight that, unlike conventional modifiers such as graphene or AgNPs, which are prone to aggregation and often require binders for electrode immobilization, the MOF provides a stable structure with uniformly distributed catalytic sites.^55,56^ This feature not only facilitates reproducible fabrication but also contributes to stable baselines and reduced signal variability, which are essential for consistent analytical performance. Collectively, these attributes emphasize the novelty of employing [Cu­(C_8_H_4_O_4_)]_*n*_ as an electrode modifier and emphasize its clear advantages over conventional nanomaterials. These findings demonstrate that the sensor efficiently detects CIP at trace levels, establishing it as a promising tool for accurately quantifying low concentrations in complex matrices, including food and biological samples.

In addition to the electrochemical sensors summarized in Tables 3 and S1 presents representative chromatographic methods reported for the determination of CIP in egg samples. Techniques such as high-performance liquid chromatography (HPLC), ultraperformance liquid chromatography-tandem mass spectrometry (UPLC-MS/MS), and liquid chromatography coupled with quadrupole/orbitrap high-resolution mass spectrometry (LC-Q/Orbitrap-HRMS) are well established and typically achieve low LODs with high analytical accuracy^57^ (Table S1).

Nonetheless, chromatographic techniques generally involve labor-intensive sample preparation, require expensive instrumentation, and are associated with longer analysis times. Thus, while chromatographic approaches offer superior sensitivity and selectivity, electrochemical sensors represent a complementary alternative that is faster, more cost-effective, and environmentally friendly, although sometimes with slightly higher detection limits.^58^ Therefore, both approaches can be considered complementary, with the choice depending on the analytical context, the available resources, and the required sensitivity.

## 4 Conclusion

In conclusion, the mesoporous copper coordination polymer, [Cu­(C_8_H_4_O_4_)]_*n*_, was successfully synthesized and characterized, demonstrating its potential for electrochemical applications. When employed as a modified electrode, [Cu­(C_8_H_4_O_4_)]_*n*_/CPE enabled sensitive and selective detection of CIP, providing consistent analytical performance. The electrochemical sensor exhibited good performance in synthetic urine samples, reaching a LOD of 0.5 μmol L^–1^ and LOQ of 1.7 μmol L^-–1^. For eggs white samples, the LOD and LOQ were determined to be 3.0 μmol L^–1^ and 10.1 μmol L^–1^, respectively. Furthermore, the recovery rates ranged from 78.76 to 108.30%, indicating satisfactory accuracy for the determination of CIP in complex matrices, with a relative RSD below 10.16% (*n* = 3). These results confirm the suitability of the proposed sensor as an efficient platform for CIP detection with high accuracy.

## Supplementary Material



## Notes

Additional experimental data that complement the findings presented in the main text; figures related to the optimization of the electrochemical detection of ciprofloxacin using the modified electrode (PDF)
