# Supplementary material for: Integration of Mesoporous Copper Coordination Polymer in Electrochemical Platform for Detection of Fluoroquinolone Antibiotic in Biological and Food Samples
Source: ACS Omega. 2025 Oct 21;10(43):51466–78. doi: 10.1021/acsomega.5c07161 (PMC12594003; doi:10.1021/acsomega.5c07161)
Supplement: Supplementary file 1 [file ao5c07161_si_001.pdf]

Supplementary Material

Integration of Mesoporous Copper Coordination Polymer in Electrochemical Platform for Detection of Fluoroquinolone Antibiotic in Biological and Food Samples

Iare S. Ribeiro[a], Tatianny de A. Andrade[a], Tiago A. Silva[b], Jemmyson R. de Jesus[a*]

[a] Research Laboratory in bionanomaterials, LPbio, Department of Chemistry, Federal University of Viçosa, 36570-900 Viçosa, Minas Gerais, Brazil.

[b] Department of Chemistry, Federal University of Viçosa, 36570-900 Viçosa, Minas Gerais, Brazil.

*Corresponding author:

Prof. Jemmyson R. de Jesus

E-mail address: jemmyson.jesus@ufv.br

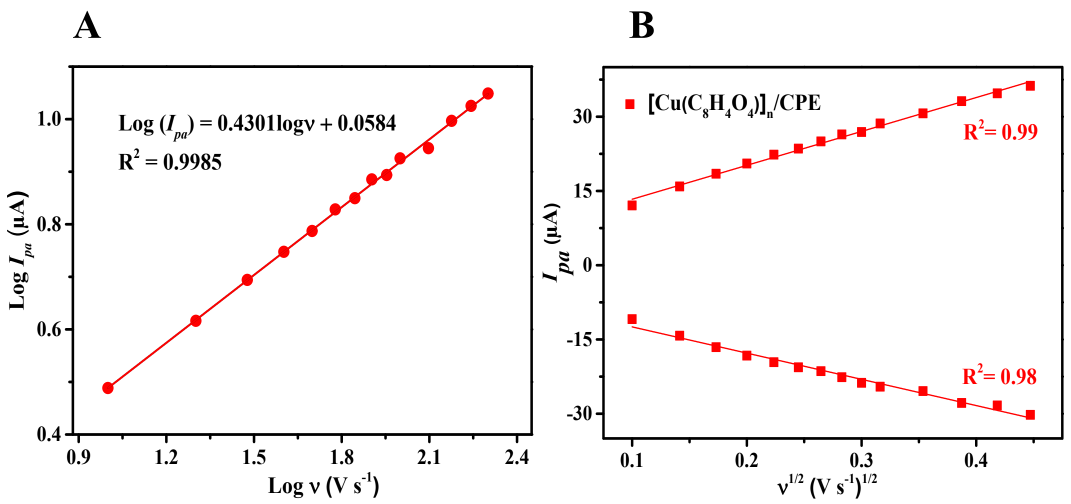

Figure S1. Electrochemical behavior of the $Fe(CN)6^{3-/4-}$ redox. **(A)** plot of log I_{pa} vs. log ν and (**B**) plot of I_{pa} vs. $\nu^{1/2}$. All measurements were performed using the 20%-[Cu($C_8H_4O_4$)]$_n$ /CPE electrode in 0.1 mol L^{-1} phosphate buffer solution (pH 6) containing 5.0×10^{-4} mol L^{-1} of $Fe(CN)6^{3-/4-}$.

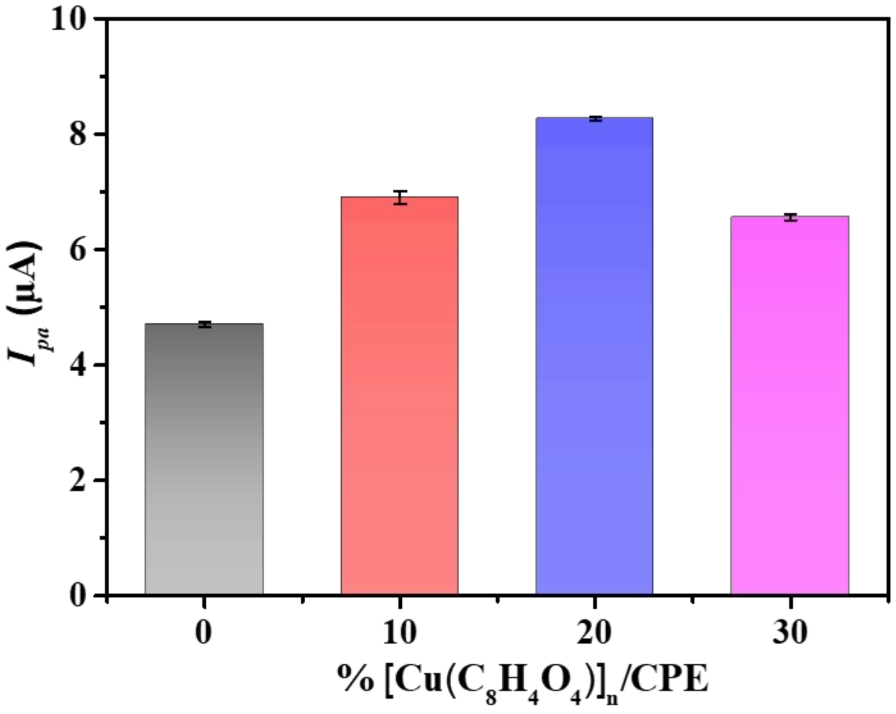

Figure S2. Graph illustrating the relationship between anodic peak current (I_{pa}) of CIP (44.3 μmol L^{-1}) and with different percentages of the [Cu($C_8H_4O_4$)]$_n$ (10, 20, and 30%, w/w) in a phosphate buffer (0.1 mol L^{-1}, pH 6) at a scan rate of 50 mV s^{-1}.

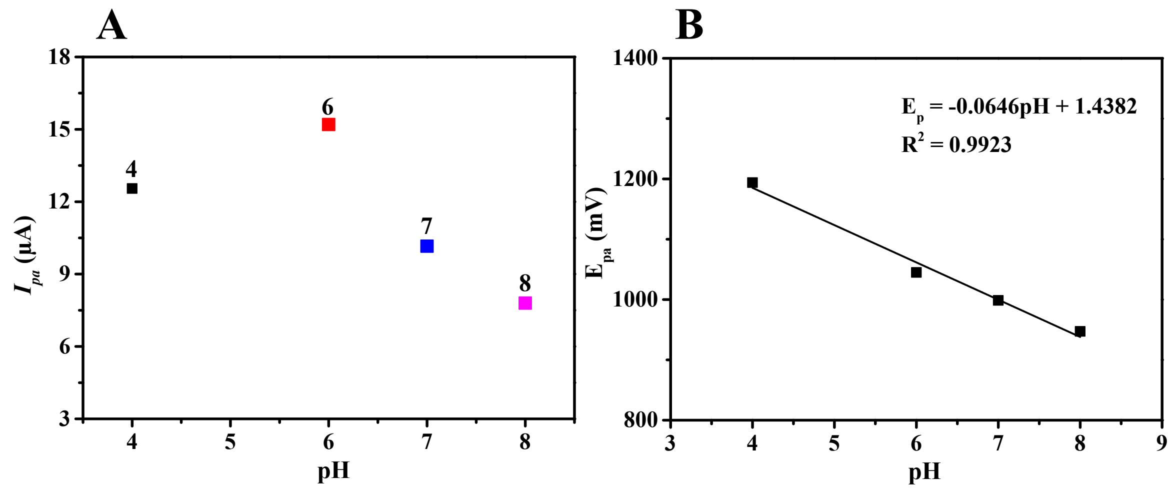

Figure S3. (A) Relation between pH and anodic peak current (I_{pa}) and **(B)** linear plot of anodic peak potential (E_{pa}) of ciprofloxacin (CIP) versus pH obtained for CIP.

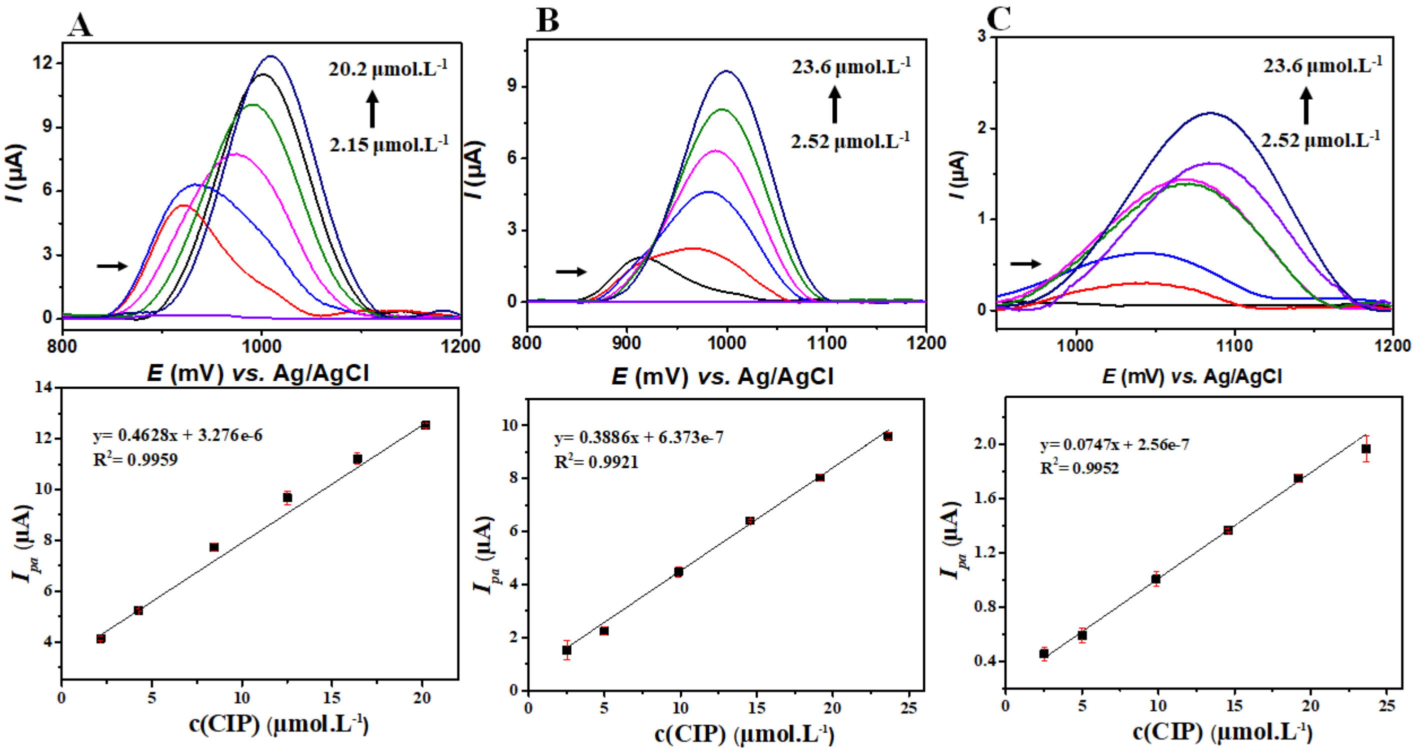

Figure S4. Differential pulse voltammograms (DPV) recorded using 20%-$[Cu(C_8H_4O_4)]_n$/CPE for varying concentrations of CIP: **(A)** From 2.15 μmol L^{-1} to 20.2 μmol L^{-1} in phosphate buffer solution (0.1 mol L^{-1}, pH 6.0) and analytical curve obtained for CIP (I_{pa} vs. c(CIP)) in phosphate buffer solution; **(B)** From 2.52 μmol L^{-1} to 23.6 μmol L^{-1} in synthetic urine and analytical curve obtained for CIP (I_{pa} vs. c(CIP)) in the synthetic urine sample; **(C)** From 2.52

μmol L^{-1} to 26.6 μmol L^{-1} in egg white diluted into the phosphate buffer solution at a ratio of 1:500 and analytical curve obtained for CIP (I_{pa} vs. c(CIP)) in the egg white sample.

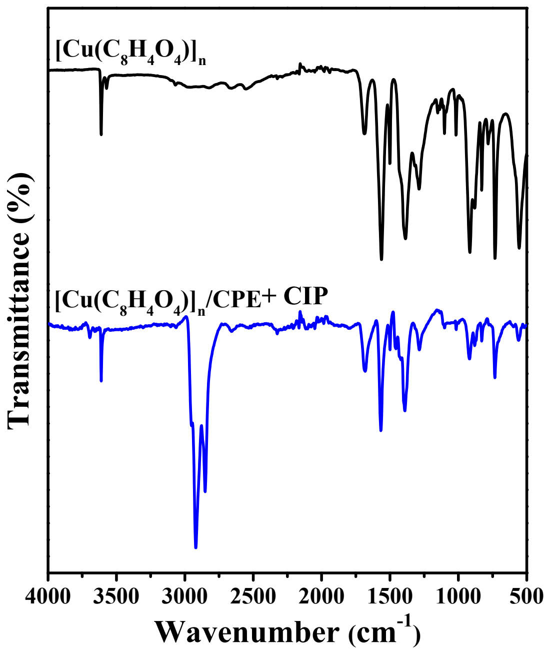

Figure S5. FT-IR spectra of $[Cu(C_8H_4O_4)]$ before and after electrochemical detection of ciprofloxacin (CIP).

Table S1. Representative chromatographic methods for ciprofloxacin (CIP) determination in eggs and other matrices.

Method	Sample	Linear range	LOD	LOQ	Recovery (%)	Ref.
HPLC	Eggs	0.14-0.28 μg g^{-1}	0.16 μg g^{-1}	NS	49-85	[74]
MSPE-UPLC-MS/MS	Eggs	0.2–200 (ng mL $^{-1}$)	0.15 µg L^{-1}	0.51 µg L^{-1}	70–119	[75]
LC-Q/Orbitrap-HRMS	Eggs	1.0~10.0 ng mL^{-1}	0.12 ng mL^{-1}	NS	more than 80	[76]
MSPE-UHPLC-MS/MS	Eggs	0.10–100 µg kg^{-1}	0.05 µg kg^{-1}	0.10 µg kg^{-1}	75-103	[77]

HPLC: high performance liquid chromatography; **LC-Q/Orbitrap-HRMS:** liquid chromatography coupled to quadrupole/orbitrap high-resolution mass spectrometry; **MSPE-UHPLC-MS/MS:** magnetic solid phase extraction coupled with ultra-high-performance liquid chromatography–tandem mass spectrometry. **MSPE-UPLC-MS/MS:** magnetic solid phase extraction coupled with ultra-performance liquid chromatography–tandem mass spectrometry; **NS:** not specified.
